# Supplementary material for: Reference values for respiratory muscle strength and maximal voluntary ventilation in the Brazilian adult population: A multicentric study
Source: PLoS One. 2024 Nov 21;19(11):e0313209. doi: 10.1371/journal.pone.0313209 (PMC11581219; doi:10.1371/journal.pone.0313209)
Supplement: S1 Table — (DOCX) [file pone.0313209.s001.docx]

**SUPPLEMENTARY MATERIAL**

S1. Description of prediction equations for MRP and MVV from previous studies.

|  | | **Present study** | **Pessoa et al (2014)** | **Costa et al (2010)** | **Simões et al (2010)** | **Neder et al (1999)** |
| --- | --- | --- | --- | --- | --- | --- |
| **Sample (n)** | **M** | 111 | 60 | 60 | 70 | 50 |
|  | **W** | 132 | 74 | 60 | 70 | 50 |
| **Age (year)** |  | 20 to 80 | 20 to 65 | 20 to 80 | 20 to 89 | 20 to 80 |
| **Location (state)** |  | Rio Grande do Norte; São Paulo; Pernambuco | Minas Gerais | São Paulo | São Paulo | São Paulo |
| **Device** |  | Digital manometer MicroRPM | Digital manometer NEPEB-LabCare | Aneroid manometer GER-AR | Aneroid manometer GER-AR | Aneroid manometer Gauger |
| **Physical activity (inclusion criteria)** |  | Baecke questionnaire (2003) | ACSM recommendation (2007) | - | Baecke questionnaire (2003) | Baecke questionnaire (1982) |
| **Pulmonary function (inclusion criteria)** |  | Spirometry values | Spirometry values | - | - | - |
| **BMI (inclusion criteria)** |  | 18.5 to 29.9 Kg/m^2^ | 18.5 to 29.9 Kg/m^2^ | 18.0 to 29.5 Kg/m^2^ | 18.5 to 29.9 Kg/m^2^ | 18.5 to 39.9 Kg/m^2^ |
| **MIP (equation)** | **M** | 137 – 0.57 x (age) | 63.27 – 0.55 x (age) + 17.96(gender) + 0.58(weight) 1 for men and 0 for women | 149.33 – 1.14 x (age) | 125 – 0.76(age) | 155.3 – 0.80 x (age) |
|  | **W** | 107.3 – 0.4 x (age) |  | 74.25 – 0.46 x (age) | 80.7 – 0.85 x (age) – 0.3(weight) | 110.4 – 0.49 x (age) |
| **MEP (equation)** | **M** | 179.9 – 0.67 x (age) | 2.29 x (age) – 0.03 x (age^2^) + 33.72 x (gender) + 1.40 x (waist) –61.41 1 for men and 0 for women | 183.31 – 1.26 x (age) | 87.69 – 0.83 x (age) | 165.3 – 0.81 x (age) |
|  | **W** | 127.4 – 0.43 x (age) |  | 119.35 – 0.68 x (age) | 125.1 – 0.89 x (age) – 0.18 x (weight) | 115.6 – 0.61 x (age) |
| **MVV (equation)** | **M** | 206.3 – 1.18 x (age) | - | - | - | 199.1 – 1.12 x (age) |
|  | **W** | 146.3 – 0.86 x (age) | - | - | - | 147.4 – 0.76 x (age) |

BMI: body mass index; MIP: maximal inspiratory pressure; MEP: maximal expiratory pressure; MVV: maximal voluntary ventilation; M: men; W: women.
