# Supplementary material for: Reference values for respiratory muscle strength and maximal voluntary ventilation in the Brazilian adult population: A multicentric study
Source: PLoS One. 2024 Nov 21;19(11):e0313209. doi: 10.1371/journal.pone.0313209 (PMC11581219; doi:10.1371/journal.pone.0313209)
Supplement: Attachment — Submitted filename: Response to Reviewers.pdf [file pone.0313209.s003.pdf]

Universidade Federal do Rio Grande do Norte

PneumoCardioVascular Lab

Departamento de Fisioterapia

Centro de Ciências da Saúde - UFRN

Campus Universitário Lagoa Nova

Caixa Postal 1524 - CEP:59072-970-Natal-RN

Fone: (84)33422002

Response to Reviewers

April 22, 2024

Dear Editor,

We appreciate for reviewing our paper and providing valuable comments. We have carefully considered each comment and have made revisions to the manuscript accordingly. We are confident that these revisions have addressed the concerns raised and have improved the overall quality of the manuscript.

Below, we provide a point-by-point response to the reviewers' comments:

Correction of Data Collection Dates:

Thank you for bringing this to our attention. We have corrected the error in the initial date of data collection. The correct dates are as follows: September 01, 2009, and December 25, 2011 (Page 4, lines 90-91).

We believe that these revisions have strengthened the manuscript and hope that it now meets the high standards for publication in your esteemed journal.

Thank you for your consideration.

Sincerely,

Guilherme A F Fregonezi

Corresponding author

Email: guilherme.fregonezi@ufrn.br Tel.: +55 84 994267896.
